# Adapting to the Airways: Metabolic Requirements of *Pseudomonas aeruginosa* during the Infection of Cystic Fibrosis Patients

**DOI:** 10.3390/metabo9100234

**Published:** 2019-10-16

**Authors:** Ruggero La Rosa, Helle Krogh Johansen, Søren Molin

**Affiliations:** 1 The Novo Nordisk Foundation Center for Biosustainability, Technical University of Denmark, 2800 Kgs. Lyngby, Denmark; 2 Department of Clinical Microbiology 9301, Rigshospitalet, 2100 Copenhagen, Denmark; 3 Department of Clinical Medicine, Faculty of Health and Medical Sciences, University of Copenhagen, 2200 Copenhagen, Denmark

**Keywords:** metabolism, adaptation, evolution, cystic fibrosis, *Pseudomonas aeruginosa*

## Abstract

*Pseudomonas aeruginosa* is one of the major causes of morbidity and mortality of cystic fibrosis patients. During the infection, the bacteria colonize the nutritional rich lung mucus, which is present in the airway secretions in the patients, and they adapt their phenotype accordingly to the lung environment. In the airways, *P. aeruginosa* undergoes a broad metabolic rewiring as a consequence of the nutritional and stressful complexity of the lungs. However, the role of such metabolic rewiring on the infection outcome is poorly understood. Here, we review the metabolic evolution of clinical strains of *P. aeruginosa* during a cystic fibrosis lung infection and the metabolic functions operating in vivo under patho-physiological conditions. Finally, we discuss the perspective of modeling the cystic fibrosis environment using genome scale metabolic models of *P. aeruginosa*. Understanding the physiological changes occurring during the infection may pave the way to a more effective treatment for *P. aeruginosa* lung infections.

## 1. Introduction

*Pseudomonas aeruginosa* is an opportunistic pathogen with high medical importance. It is commonly found in natural environments such as soils, plants, and waters but it can also cause diseases in plants, animals, and humans [1]. The World Health Organization (WHO) included it in the list of critical priorities due to its infecting capabilities and its multidrug resistance [2]. In Cystic Fibrosis (CF) patients, *P. aeruginosa* causes long-term chronic airways infections, often lasting for more than 30 years. CF is caused by mutations in the Cystic Fibrosis Transmembrane Conductance Regulator protein (CFTR) affecting the chloride ion channel activity. Such mutations, leading to an unfunctional or misfolded CFTR transporter, cause dysregulation of the epithelial fluid transport with accumulation of large amount of sticky and dehydrated mucus in the lungs [3]. While in healthy people the mucus provides protection against inhaled particles and it is easily cleared through the mucociliary escalator, its abnormal accumulation in CF patients causes breathing difficulties, persistent coughing and lung inflammation such as bronchitis and infective pneumonia. Over time, this condition leads to reduced lung function which will eventually lead to lung transplantation as a consequence of the progressive respiratory failure. A detailed review of CF pathogenesis can be found in [4]. Since it contains variegated and plentiful nutrients, the CF mucus constitutes an optimal substrate for bacterial growth [5]. *P. aeruginosa* has a broad metabolic repertoire, which allows it to grow under different environmental and physiological conditions by employing its variegated catabolic and anabolic pathways [6]. Even though CF patients are routinely treated with antibiotics to suppress the infections, high bacterial fitness and resistance to antibiotics contribute to long-term chronic infections [7]. Metabolism is the currency of the cell and during the infection it is rewired to accommodate the needs of the cell according to the environment, the nutrients, and the stresses present in the CF airways [8]. Loss of metabolic functions, caused by mutations accumulated during the infection, has been proposed to be of selective advantage in the CF environment. Indeed, *P. aeruginosa* has a high capability of adapting according to the changing conditions and the presence of stresses. This makes it successful in colonizing the CF airways for long time periods and difficult to treat in the clinics. While much attention has been focused on *P. aeruginosa* genotypic and phenotypic adaptation, resistance to antibiotics and pathogenesis, the link between metabolic potential, metabolic adaptation, and a successful infection is still unclear. Here we review the composition of the CF mucus, the metabolic reorganization of *P. aeruginosa* during evolution in CF patients and the metabolic functions active in the CF lung environment. Lastly, we discuss if metabolic models can contextualize the observed metabolic changes to model the CF environment and *P. aeruginosa* lung infection.

## 2. The Cystic Fibrosis Mucus

### 2.1. Individual Complexity of the CF Mucus

**Figure 1:**
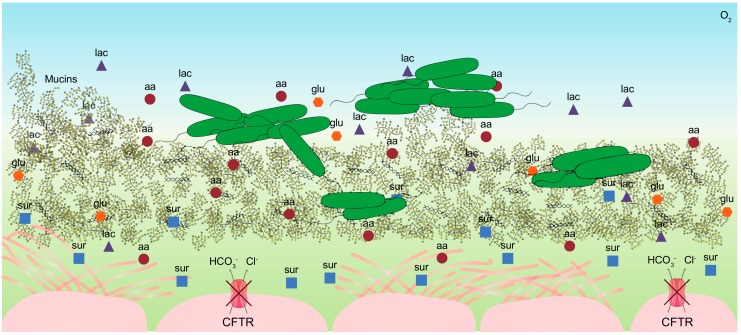
The cystic fibrosis airway environment. The figure (not to scale) is a schematic representation of the environment in which *Pseudomonas aeruginosa* grows which includes nutrients such as amino acids (aa), lactate (lac), glucose (glu), surfactants (sur), DNA, mucins, and gradients of oxygen (O_2_).

The CF mucus is a rich complex substrate which provides carbon and energy sources for high density growth of *P. aeruginosa* during the infection [5]. A schematic presentation of the CF airway environment is shown in Figure 1. The abnormal mucus accumulation in the CF lungs is due to imbalanced ion transport trough the CFTR channel which produces a hyper-concentrated substrate which strongly adheres to the airway surfaces [3]. The mucus contains high concentrations of mucins, DNA, free amino acids, glucose, lactate, and ions, which make the substrate highly viscous and very difficult to analyze using standard chemical and metabolomics techniques [9]. In 2007, Palmer et al. analyzed 12 sputum samples from adult CF patients with non-exacerbating *P. aeruginosa* resulting in a detailed description of the CF mucus composition [10]. Recently, analyses of metabolomes of longitudinal sputum samples from 11 patients during exacerbations (exacerbation, treatment, post-treatment, or stable) showed that the individual patients constitute a greater source of variation in the metabolome composition than the clinical state of the patient [11]. Indeed, the specific metabolite diversity, the total amino acid content and the concentration of each metabolite vary between samples, which therefore reveal a high divergence in the mucus composition between patients [10,11]. Moreover, metabolites, stresses, and antibiotics are not evenly distributed in the distinct regions of the lungs contributing to high niche complexity [12]. This underlines the individual complexity of the CF environment, which in turn leads to high bacterial diversity and heterogeneous trajectories of bacterial evolution [13].

### 2.2. Composition of the CF Sputum

The CFTR mutation causes unbalanced ion concentrations in the mucus, which can reach around 70 mM in the case of Cl^−^ and Na^+^ [10]. This disequilibrium makes the CF secretions hyper-saline. Amino acids are one of the most available nutrients for *P. aeruginosa*. Moreover, they can be directly used as building blocks for biomass production or catabolized to generate reducing equivalents [14,15]. The concentration of amino acids in CF sputum, in the range of 5–25 mM, allows high growth rate and high cellular biomass [5,10]. Analysis of sputum samples from CF and non-CF patients showed that all amino acids are present in both samples, but CF sputum contains higher concentrations than non-CF [16]. Glucose is also present in CF and non-CF mucus. The concentration can vary greatly from patient to patient and during distinct clinical stages, even though the concentration is generally higher in CF than other pulmonary diseases [10,16,17]. However, although glucose is present in the CF mucus (range 1.3–4.5 mM) [10], it is not a preferred carbon source for *P. aeruginosa*, which preferentially assimilate amino acids and organic acids over sugars [14]. The CF mucus contains between 3.6 and 15 mM lactate that is likely secreted by neutrophils during anaerobic growth typical of the CF mucus in the lungs [18]. Similarly, as a consequence of inflammation, neutrophils release high molecular weight DNA (range 0–9.5 mg/mL), which increases the viscosity of the airways secretions [19,20]. Mucins are high molecular weight glycoproteins, which form a discontinuous layer in the airways contributing to protection of the airway epithelium, providing defense against inhaled particles and invading bacteria. Moreover, they contribute to the high viscosity and the structural complexity of the CF mucus [21]. Their concentration range between 0.1 and 5.7 mM and can be up to 100-fold higher than the DNA in sputum [22,23]. Even though mucins are an available source of both carbon and nitrogen in the mucus, *P. aeruginosa* is unable to catabolize this nutrient [24]. However, while mucins may not directly contribute to growth of *P. aeruginosa* in the CF lungs, they do promote diversification in lung-like conditions underlining the role of nutritional and structural complexity in driving evolution [25]. Pulmonary surfactants, which consist of a mixture of lipids and proteins secreted by the epithelial cells, play a crucial role in reducing the surface tension at the air/liquid interface in the lungs, which helps to avoid alveolar collapse at the end of expiration and to ease the breathing [26]. Analysis of the bronchial surfactant documented a significant heterogeneity of the chemical composition and distribution in different CF patients. However, in all cases the results pointed to an altered and dysfunctional composition of the surfactant component due to lung injuries in CF patients [27,28,29]. Oxygen is often available for *P. aeruginosa* at limiting concentrations, due to the presence of thick and viscous mucus in the lungs and to the consumption by immune cells and other members of the lung microbiota. Direct oxygen measurements in situ using O_2_ sensors detected steep oxygen gradients (180–2.5 mmHg) in the mucus, which contribute to micro-aerophilic or even anaerobic conditions [30]. *P. aeruginosa* can grow both micro-aerobically and anaerobically using nitrate (NO_3_^−^) or nitrite (NO_2_^−^) as the final electron acceptors in the electron transport chain and using arginine for fermentation. Nitric oxide metabolites are widely available in CF sputum (346 µmol/L - 95% CI 311–504) and their concentration is more than twice that of sputum from healthy individuals [31,32]. These conditions of reduced oxygen availability induce drastic metabolic changes in *P. aeruginosa*, which result in increased antibiotic tolerance, production of virulence factors, biosynthesis of alginate and biofilm development, ultimately leading to a chronic infection [33]. 

### 2.3. Mimicking the Nutritional Lung-Like Conditions

The need for a medium to recreate the CF environment in vitro, and for studying the physiology of *P. aeruginosa* in lung-like conditions, has led the development of the Synthetic CF Medium (SCFM2) and the Artificial Sputum Medium (ASM), that both mimic the CF sputum composition [10,34,35]. Even though the specific formulations vary between SCFM2 (a defined medium) and ASM (an undefined medium), both contain all the fundamental components of the CF mucus, and they represent the gold standards used in many laboratories for investigating *P. aeruginosa* in a CF context. *P. aeruginosa* displays similar phenotypes during growth in CF sputum and in SCFM, including similar growth rates and gene expression profiles, carbon substrate preferences and cell-to-cell signaling profile [10,35]. However, although it is thus possible to mimic the nutritional lung-like conditions in vitro, it is still largely unexplored how the composition and concentration of distinct metabolites are spatially distributed in the mucus, how conditions vary from patient to patient, and to what extent these factors contribute as drivers for evolutionary trajectories, which lead to chronic infections [12].

## 3. Diversity of Metabolic Potential over Infection Time

### 3.1. Heterogeneity of Metabolic Profiles

**Figure 2:**
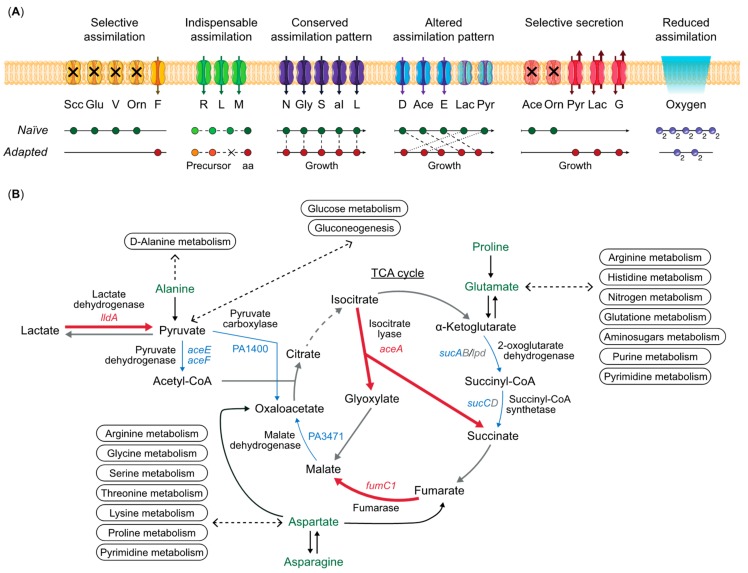
Metabolic reorganization of *Pseudomonas aeruginosa* during patient evolution. (**A**) The figure represent the metabolic configuration of adapted strains compared to naïve strains where metabolites show: selective assimilation (succinate, glucose, valine, and ornithine); indispensable assimilation due to auxotrophy (arginine, leucine, and methionine); conserved assimilation pattern (asparagine, glycerol, serine, alloisoleucine, and leucine); altered assimilation pattern (aspartate, acetate, glutamate, lactate, and pyruvate); selective secretion (acetate, ornithine, pyruvate, lactate, and glycine); reduced assimilation (oxygen) [8]. A detailed description of the changes in metabolic profiles during evolution is presented in paragraph 3. (**B**) In vivo metabolic program of *P. aeruginosa*. Genes upregulated in vivo relative to in vitro are represented in red, genes downregulated in blue, and genes which expression is constant in both conditions are in gray [61]. Compounds assimilated during exponential phase by adapted clinical strains of *P. aeruginosa* are indicated in green [8]. The dotted lines represent related metabolic pathways connected to the assimilated compounds.

*P. aeruginosa* has a wide metabolic repertoire, which allows it to colonize different environments [6]. Although this broad metabolic capacity together with a broad range of virulence factors allow the bacteria to colonize different niches, the environmental conditions and the availability of nutrients may limit the use of metabolic pathways to fit the local needs. Nutrient diversity and availability vary between the lungs and the environment, reshaping the *P. aeruginosa* metabolic profile during the infection. Analysis of the metabolic repertoire of clinical strains of *P. aeruginosa* has shown a broad metabolic profile heterogeneity, underlining the importance of spatial and chemical diversity in the environment during evolution in the CF lungs [36,37,38]. This metabolic heterogeneity is accompanied by a broader phenotypic and genotypic heterogeneity, which is maximized during the first years of infection, when adaptive evolution results in reshaping the bacterial physiology in response to the CF environment [13,39]. In spite of this heterogeneity, parallel changes in bacterial metabolism have been observed in longitudinal studies, identifying dominant characteristics of long-term adaptive evolution. A schematic representation of the changes in metabolism of *P. aeruginosa* during evolution in the CF environment is presented in Figure 2A.

### 3.2. Amino Acids Auxotrophies

Auxotrophy is the inability of synthetizing one or more specific metabolites required for growth, which frequently is observed when such metabolites are widely available in the environment. Amino acids are valuable carbon sources for *P. aeruginosa* and the CF mucus is rich in these metabolites. Analyses of more than 200 sputum samples from 60 CF patients with reduced lung function showed that in more than 60% of the patients the increased amino acids concentration promoted the development of auxotrophy from pre-existing prototrophic bacteria [40,41]. Methionine, leucine, and arginine auxotrophies are frequently observed among clinical CF isolates of *P. aeruginosa*, and related non-synonymous mutations in the biosynthetic pathways for these amino acids have been identified [8,42]. Since amino acid biosynthesis is metabolically ‘expensive’ [43], bacterial loss of the ability to synthetize them when they are available in the environment, creates a positive selective advantage of auxotrophic over prototrophic strains [44]. However, while auxotrophy contributes to high fitness in the CF airways, it limits the ability of *P. aeruginosa* to move back to environments, where nutrient availability is often limited. 

### 3.3. Metabolic Rewiring of Clinical Strains

Biolog Phenotype MicroArrays (PMs) are excellent tools to determine global metabolic profiles of bacteria, and they have also been used for clinical strains of *P. aeruginosa*. Several such investigations have concluded that there is a general reduction of the catabolic repertoire in clinical isolates during the time of infection [45,46]. Analysis of 35 clinical strains longitudinally isolated from 10 CF patients during intermittent colonization and chronic lung infection showed that the extensive heterogeneity in the metabolic profiles observed in early stage isolates is clearly reduced in isolates from later stages [38]. Substrates such as alanine, asparagine, glutamine, arginine, and proline showed the highest reduction in the catabolic ability over infection time. It is important to note, however, that the PM method is based on analysis of bacterial catabolism in presence of single substrates. This means that when observing loss or reduction of the catabolic repertoire, it may be difficult to distinguish between an actual compromised catabolic pathway, or an impaired unrelated pathway caused for example by auxotrophy [8,42].

The CF mucus is a complex substrate, requiring more comprehensive metabolomics approaches to investigate bacterial metabolic evolution during adaptation in the patient. In a study of 179 strains isolated from 18 CF patients over a 24 years period following the first *P. aeruginosa* isolation, supernatant samples from these strains grown in lysogenic broth (LB) were analyzed using Nuclear Magnetic Resonance (NMR) untargeted metabolomics [37]. Even though only a single supernatant sample after 24 hours of growth was analyzed per strain, this approach resulted in the identification of 32 metabolites assimilated or secreted by *P. aeruginosa*. Moreover, the obtained data could be used to determine a negative correlation between the concentration of seven metabolites and the length of infection. As an example, acetate was observed to be secreted at higher concentrations from isolates with short infection age compared with long-term adapted strains. Amino acids such as phenylalanine, tryptophan, tyrosine, valine, lysine, and serine showed either reduced excretion or increased consumption over time. These results suggest that CF clinical strains evolve towards a more complete utilization of metabolically costly compounds relative to other compounds [37]. However, strains isolated from distinct patients showed the highest degree of variability in metabolite levels, in accordance with an individual composition and structure of the CF lung environment and consequential distinct trajectories of within-patient evolution [11,37].

To characterize the metabolic specialization developing during long-term infection, six clinical isolates of *P. aeruginosa*, representing different stages of evolution (naïve, intermediate, and adapted), were isolated from sputum samples collected from one CF patient over a period of 8 years of infection [8]. Using a metabolic footprinting approach [47,48], where multiple supernatant samples were collected during the growth in LB medium and analyzed by targeted metabolomics, a time-dependent map of the metabolic requirement and preference of the strains was created. When compared with naïve strains, the adapted strains showed: (1) auxotroph phenotypes due to mutations in biosynthetic genes for amino acids; (2) a preference of assimilation of carbon sources distinct from the one of naïve strains designed for the CF lung resources; (3) reduced arginine and pyruvate fermentation processes; (4) reduced acetate secretion; and (5) increased oxygen requirement [8]. A schematic representation of the metabolic behavior of naïve strains compared with adapted strains of *P. aeruginosa* is shown in Figure 2A. In brief, analysis of the assimilation and secretion patterns showed that assimilation of four metabolites was reduced in the adapted strains, suggesting selective assimilation. Three amino acids were indispensable substrates for growth of the adapted strains due to auxotrophy. Assimilation of five metabolites was conserved, since no differences in the assimilation patterns between naïve and adapted strains were observed. Altered assimilation patterns in the adapted strains for five amino acids and organic acids were shown as differential substrate uptake during the exponential and stationary phases according to the degree of adaptation. Due to different distributions of metabolic fluxes and fermentation processes, naïve and adapted strains showed selective secretion profiles for five metabolites. The assimilation of oxygen, the concentration of which fluctuates in the CF sputum, was reduced in adapted strains (Figure 2A) [8]. While both the naïve and the adapted clinical isolates maintained their metabolic profiles during 8 years of adaptation in the patient, the intermediate strains showed both characteristics, indicating transition from the naïve to the adapted stage. This work shows how the adaptive changes occurring during evolution in the CF environment support the idea of parallel evolution of metabolism. However, attempts to align the metabolic profiles with the mutations identified in the genomes failed to identify causality for the observed phenotypes due to high numbers of mutations and the complexity of the regulatory networks governing metabolism [8]. 

### 3.4. Mutations on Non-Metabolic Genes Causing Metabolic Changes

Many mutations accumulated in *P. aeruginosa* genomes during infections in the airways of CF patients have been characterized both molecularly and phenotypically [49,50]. However, the metabolic costs and consequences of most mutations are largely unknown. In fact, it is unclear if metabolic adaptation is specifically selected for in vivo, or if it indirectly develops as consequence of other unrelated mutations. Metabolism is highly regulated and changes in distant regulatory networks or changes in the expression of metabolic enzymes can have a broad metabolic impact independently of mutations in metabolic genes [15]. One of the main regulators of the *P. aeruginosa* quorum sensing system (QS) – LasR – activates the expression of many virulence factors [51,52,53]. The *lasR* gene is under strong selective pressure, and many mutations in this gene have been identified in clinical strains from both young and adult patients [39,53,54,55,56]. LasR mutants undergo a dramatic metabolic shift associated with a growth advantage in presence of specific amino acids and nitrate as nitrogen source, which are both abundant in the CF mucus. Moreover, LasR mutant strains show decreased oxygen consumption and increased tolerance to tobramycin and ciprofloxacin, which are routinely used for the treatment of *P. aeruginosa* infections in CF patients [55,56]. Similarly, inactivation of both the *lasR* and *rhlI* genes, responsible for the production of two of the most important QS signals in *P. aeruginosa*, causes a global metabolic rearrangement of the cell with changes in the cellular exo-metabolome, perturbations in the tricarboxylic acid (TCA) cycle intermediates, amino acids, and fatty acids metabolites, resulting in reorganization of the cellular metabolic fluxes [57]. Increased antibiotic resistance caused by overexpression of multidrug-resistant (MDR) efflux pumps is an important survival strategy for *P. aeruginosa* in the CF environment [58]. In the case of the MexEF-OprN, MexAB-OprN, MexCD-OprJ, and MexXY pumps, it has been shown that to compensate for the fitness cost of overexpressing these costly transporters, bacteria increase nitrate consumption and enhance oxygen uptake to counterbalance the intracellular H^+^ accumulation [59,60]. Even though the examples above have mainly been reported for *P. aeruginosa* laboratory strains, they all suggest that rewiring of metabolism may occur as a compensatory mechanism for metabolically unrelated changes in other traits. 

In conclusion, it is still unclear if bacterial metabolic adaptation should be seen as a response to nutrient availability within the host environment, or if it is an indirect fitness consequence of mutations in genes responsible for unrelated phenotypes.

## 4. *Pseudomonas aeruginosa* In-Situ Metabolic Program

### 4.1. In Vitro vs. in Situ Expression Profiles

Adaptive processes occurring in the CF lung environment using whole genome sequencing (WGS) of strains isolated from CF patients have been described previously [39,62,63]. Pathoadaptive mutations in evolutionary important genes have been characterized in clinical isolates from longitudinal collections of *P. aeruginosa* [39]. This pattern of genomic convergence, however, has been challenged by extensive phenotypic diversity, which has limited our ability to predict specific outcomes of mutations [13]. Moreover, different lineages showing individual genomic patterns often display similar phenotypes or similar expression profiles, independently of the accumulated mutations [46,61,64,65,66]. These observations create severe challenges for predictions of infection outcome based on genome sequencing. 

To overcome some of these problems, direct investigations of bacterial expression profiles in sputum samples from individual patients have been used to obtain insight in the in situ lifestyle of *P. aeruginosa* in the host. Due to the characteristics of the CF mucus, the dynamics of the CF airway environment, and the interference from host cells, only few conclusive results are available [61,67,68,69]. Both RNA-seq and proteomics based approaches have been employed to provide snapshots of the bacterial metabolic profiles in the lungs of CF patients. For relative quantification of specific RNAs and proteins in sputum samples, comparisons of the expression profiles in situ (metatranscriptome or metaproteome) with the expression profiles in vitro in laboratory conditions of isolated strains from the same sputum sample were carried out. In one investigation, the global gene expression profiles (patho-phenotypes) of *P. aeruginosa* were determined in 15 sputum samples from five chronically infected CF patients [61], and as expected the transcriptomic signatures of the soft-core genomes in situ differed from the ones of the lab conditions [61]. Interestingly, the soft-core genome global expression profiles were surprisingly similar, independent of lineage and host specific characteristics, suggesting that adaptation to the CF lung involves a defined set of regulatory processes, which govern the core physiology and behavior of the bacterial populations with little impact from the clonal and genomic variance, the host environment and competing microorganisms. Analyses of transcriptomic profiles from five different regions of an ex vivo explanted CF lung showed reduced expression profile diversities within the individual regions independent of the genetic variation of the isolated strains [66]. Similar results were obtained when comparing proteomes of 35 sputum samples from 11 CF patients [67]. Both meta-transcriptomic and meta-proteomic investigations of very complex samples have inherent technical problems, but both identify very similar in vivo configurations of metabolism, which differ from those observed in laboratory conditions. A schematic representation of the in vivo metabolic program of the cell is shown in Figure 2B.

### 4.2. Metabolic Configuration in the CF Airways

Changes in the physiology of the cells in situ converge to a general narrowing of the cellular activity as suggested by the reduced growth rate described in vivo [70]. Even though glucose is present at high concentration in CF mucus, *P. aeruginosa* shows reduced sugar metabolism caused by the repression of the *gltBFK-oprB* genes encoding the glucose transport system [61]. Glucose is a less preferred carbon source for *P. aeruginosa* and the loss of assimilation of this compound has also been described by metabolic footprinting analysis of clinical isolates [8,14]. Moreover, reduced expression of pyruvate carboxylase and pyruvate dehydrogenase, transforming pyruvate into oxaloacetate and acetyl-CoA, respectively, suggests that *P. aeruginosa* employs a gluconeogenic rather than a glycolytic metabolic configuration (Figure 2B) [61]. Furthermore, *aceE* and *aceF*, encoding pyruvate dehydrogenase, are among the pathoadaptive genes, which are frequently mutated in clinical isolates [39]. This metabolic rewiring is further supported by an increased expression of the lactate dehydrogenase (*lldA*), which replenishes the pool of pyruvate, necessary for the gluconeogenic processes, from lactate. Proteomic analysis of *P. aeruginosa* in sputum samples showed an increase in the abundance of the *lldP* lactate permease, confirming the meta-transcriptomic results [67]. Lactate is abundant in the CF mucus, and it is used both by naïve and adapted strains, although the time of assimilation changes according to the adaptive stage [8,10]. Interestingly, in *E. coli* disruption of the pyruvate dehydrogenase complex causes resistance to certain type of chemokines with antimicrobial effects, which may also be the case in CF lungs with *P. aeruginosa* being targeted by those chemokines [71]. Expression of the genes coding for the lower part of the TCA cycle, such as oxoglutarate dehydrogenase and succinyl-CoA (*sucA*, *sucC*, and *lpd* genes), were found to be repressed in situ relative to in vitro. In contrast, isocitrate lyase (*aceA*) expression transforming isocitrate into glyoxylate was increased in situ, indicating a higher activity of the glyoxylate shunt rather than the decarboxylation steps of the TCA cycle [61]. Interestingly, expression of the C5-dicarboxilic acid transporter was increased in situ, indicating that α-Ketoglutarate, which is part of the decarboxylation steps of the TCA cycle, might be used to protect the cells from oxidative stress and cyanide poisoning, or to regulate nitrogen metabolism through the nitrogen regulator PII [61,72]. However, α-Ketoglutarate has not been identified in sputum samples [10]. Expression of fumarate hydratase (*fumC1*), which catalyzes the interconversion of fumarate and malate and modulates the redox balance, was increased in both the meta-transcriptomic and the meta-proteomic profiles, consistent with the presence of reactive (RNS) and oxidative (ROS) stresses in the lung environment [61,67,73]. Therefore, amino acids are likely carbon sources for biomass production, and the TCA cycle and the glyoxylate shunt produce ATP and the reducing equivalents (NADH and FADH_2_) necessary to counterbalance the stresses present in the lung environment. In support of the hypothesis that amino acids are the main carbon sources in vivo, genes coding for amino acid permeases (PA2202, PA2079, PA2041, and PA1916) were found to be expressed at higher levels in situ compared to laboratory conditions [61]. Moreover, analysis of ex vivo samples of an explanted CF lung showed that amino acid biosynthetic pathways were down-regulated relative to in vitro [66]. Overall, processes such as ATP synthesis coupled electron transport, cellular lipid metabolism, fatty acid biosynthesis, propionate metabolism, oxoacid metabolism, isoprenoid catabolism, oxidative phosphorylation, and fatty acid and phospholipid metabolism, were shown to be down regulated ex vivo compared to laboratory conditions, reflecting the low energy and slow growth phenotype of *P. aeruginosa* cells growing in the CF environment [66].

It is worth noting, that although transcriptomic and proteomic data are informative about relative abundances of mRNA and proteins, helping our understanding of the environmentally determined global cellular phenotypes, such data do not directly translate into metabolic fluxes. This leaves a gap between the metabolic potential of the cell and the actual cellular metabolism. Therefore, further investigations, new technologies and new approaches are needed to fully characterize the metabolic processes of *P. aeruginosa* when living in the CF mucus.

## 5. Modelling the CF Dynamics in Silico

CF is a complex genetic disorder associated with a favorable environment rich in nutrients, a diverse polymicrobial infection competing for survival, presence of antibiotics, other stresses, challenges from the host immune system, and a non-uniform distribution of all these components in different compartments of the CF airways. This scenario drives rapid evolution of *P. aeruginosa* through the CF evolutionary landscape, which creates heterogeneous solutions, multiple outcomes, and clonal diversity. While in vivo and in vitro studies are fundamental to understand and characterize *P. aeruginosa* adaptation and evolution in the patients, we are still a long way from identifying diagnostic markers useful for classification of the infection development and for predicting consequences of the infections. Can we use in silico methods to predict pathogen dominance, virulence, and antibiotic resistance profiles? Can we recreate in silico the CF environment [74]? Genome-scale metabolic network models are useful tools that allow one to simplify complex biological networks in biological and metabolic functions that can be quantified and predicted statistically. Moreover, metabolic models can predict data such as uptake and secretion rates, metabolite exchanges between species, and competing or cooperative relationships difficult to analyze in vivo [75]. 

Virulence-linked pathways and gene essentiality of *P. aeruginosa* PAO1 and PA14 have recently been modeled using genome-scale metabolic network reconstructions, which provide the most comprehensive representation of *P. aeruginosa* metabolism [76]. The models account for the function of 1129 and 1146 genes, 1495 and 1493 reactions, and 1286 and 1284 metabolites, respectively, for PA14 and PAO1 strain. Bartell et al. were able to link the contribution of metabolic genes to virulence factors, and to define the essential genes which link virulence factor synthesis and cellular growth at a systemic level [76]. These results could have great implications for targeting virulence-related pathways with respect to drug development for treatment of resistant bacteria [77]. However, it is unclear whether in silico genome-scale metabolic network models are relevant for clinical strains with hundreds of mutations and strain specific metabolic configurations. Recently, Henson et al. developed a metabolic model of CF airway communities in order to predict pathogen dominance based on the 17 most abundant bacterial taxa in sputum samples [78]. Using this approach, the authors were able to predict interactions between species mediated by competition for host-derived nutrients and cross-feeding of secreted metabolites, and to match the abundance of the different species from 16S rDNA sequencing data. Moreover, since it is not possible to analyze the uptake and secretion rates of metabolites in situ, and since many of the cross-feeding metabolites might be only present in the close proximity of the community and not detectable in sputum samples, the authors computed the uptake and secretion rates and compiled a list of additional metabolites required for the maintenance of such in silico communities [78]. However, there is considerable skepticism concerning the use of these methods, because it is still unclear if the principles for maintaining the in silico communities are valid also in vivo, and if the available metabolic models are adequate and relevant for predicting outcomes of infections.

## 6. Conclusions

CF airways are complex and highly structured environments, in which *Pseudomonas aeruginosa* is subjected to steep gradients of nutrients and to stresses of various types, and in which the bacteria optimize over time their physiology in response to the immediate surroundings. Patient to patient variations and different treatment regimens influence how adaptation of *P. aeruginosa* progresses, and which phenotypic solutions it develops. Therefore, heterogeneity is a characteristic of both the airway environment and the *P. aeruginosa* populations evolving in the patients. Moreover, bacteria with distinct phenotypic characteristics co-exists in the CF airways suggesting that metabolic adaptation may also account for competition for nutrients. For their accessibility, studies on single bacterial isolates have been extensively carried out, but they are insufficient to explain how a whole population behave in the CF airways. Indeed, social interactions between bacteria can change their metabolic profiles to account for collaboration or competition for the available nutrients [79,80]. Unfortunately, predictions on how different isolates co-habiting the same niche grow and how metabolism proceed in presence of a polymicrobial community is very difficult to evaluate both in vivo and in vitro. This leaves a gap that could be filled by the use of metabolic models that account for both population dynamics and social interactions and by extensive studies of bacterial metabolism.

However, despite the diversity of phenotypes and the many different evolutionary trajectories, phenotypic convergence occurs, suggesting that specific traits are generally selected for in vivo to increase bacterial fitness and subsequent persistent infections. Among these convergent phenotypes, reduced growth rate is a striking feature of many clinical strains, both in vivo and in vitro [8,13,37,70]. Slow growth is a phenotype with a complex causality that promotes increased resistance to antibiotics and persistence for many bacterial species [81,82]. Independently of the mechanism, a correlation between growth rate and ciprofloxacin and streptomycin susceptibility, and between growth rate and bacterial persistence, has been described for both *Escherichia coli* and *Salmonella enterica* [83,84,85]. In the CF lung environment, slow growth may be a consequence of the described metabolic specialization, but it is also likely that mutations in global regulators may cause pleiotropic effects on other unrelated traits with resulting fitness costs. Certainly, reduction of cellular metabolic activities seems to favor the infection state of *P. aeruginosa* by increasing its fitness in the CF airway environment [61,66]. Antibiotics often target central cellular processes, which are essential for growth. Mutations which alter the normal coordination of growth, as for example by disrupting central cellular processes such as replication, transcription, and translation, may trigger increased tolerance to antibiotics and a persistent phenotype showing a reduction of the metabolic functionality of the cell. 

It has been proposed to use metabolic processes as targets for next generation antibiotics and to use metabolism to resuscitate persistent cells [86]. Moreover, metabolic profiling of clinical strains may be also used as a diagnostic of the infection state. However, this requires extended in-depth investigations of the CF sputum metabolome to unfold the metabolite complexity and the differences between patients. Indeed, metabolites activity as antibiotic potentiators strongly depends on the specific metabolic profiles of clinical isolates [87]. Additional in vitro studies of cellular metabolism under in vivo like conditions can also help to clarify the metabolic specificities of clinical isolates, and new and better model systems, which recreate the physiology and complexity of the CF environment, are needed for detailed assessments of interference by new potential antagonists. A detailed review of antibiotic tolerance and metabolic adaptation can be found in [86]. Moreover, for eventual translation of research results to useful clinical applications, we need new strategies to understand and recreate in silico the CF environment with its complexity, its heterogeneity in metabolite composition, stresses, antibiotics, and bacterial species, to predict infection progression and evolutionary trajectories of the infecting microbes.
